# Supplementary material for: S Phase Duration Is Determined by Local Rate and Global Organization of Replication
Source: Biology (Basel). 2022 May 7;11(5):718. doi: 10.3390/biology11050718 (PMC9139170; doi:10.3390/biology11050718)
Supplement: Supplementary file 1 [file biology-11-00718-s001.zip › biology-1698572-supplementary.pdf]

Table S1. Known Cell Cycle Dynamics of Cells.

Cells	Organism	DNA Content (Gbp)	G1 (h)	S phase (h)	G2 or (G2/M)	Cell Cycle (h)	Source
C7-10	Mosquito (*Aedes albopictus*)		2	6	2	10	Gerenday 1996
CHO	Chinese Hamster		7	6.7	2.79 (G2/M)	16	Rex 2002
Myelocyte series	Dog	4.82		~5		16.0-18.0	Cited by Cameron&Greulich 1963
Myeloblast	Dog	4.82		~5		9	Cited by Cameron&Greulich 1963
Erythroblasts Dog Ref1	Dog	4.82	2.5	6	1.5	10	Lala P K 1966
Erythroblasts Dog Ref2	Dog	4.82	2.5	8	1.5	12	Odartchenko N 1963
Early Embryonic Drosophilla	*Drosophilla melanogaster*	0.24		0.05			Blumenthal 1974
Post MBT Embryonic Drosophilla	*Drosophilla melanogaster*	0.24		0.6			Blumenthal 1974
Kc Drosophilla	*Drosophilla melanogaster*	0.24	1.8	10	7.2	18.8	Dolfini 1970
SL-2 Drosophilla	*Drosophilla melanogaster*	0.24		14.0-16.0	6.4-8.5 (G2/M)		Rizzino 1978
Ventricular Zone	Ferret	4.82		13.3		49.4	Hutner 2015
Inner subventricular zone	Ferret	4.82		7		43	Hutner 2015
Outer subventricular zone	Ferret	4.82		6.7		48.7	Hutner 2015
Cheek pouch	Hamster		120	8.6	2.4	130	Brown J M & R Oliver 1968
Jejunum Hamster	Hamster		6	8	2	16	Lipkin M & Deschner 1968
Carcinoma (cheek pouch)	Hamster		2.4	6	2	10	Reiskin A B & R J Berry 1968
Bone marrow	Human	6.32		6.5			Cited by Cameron&Greulich 1963
Stomach	Human	6.32		18	3		Winawer S J & M Lipkin 1969

Ileum	Human	6.32		11	4		Lipkin M 1965
Colon Human	Human	6.32		13	2	60	Lipkin M 1963
Erythroblasts & Myeloblasts	Human	6.32		12			Strykmans P 1966
HeLaK	Human	9.8	8.9	9.5	3.7	22.6	Cardoso & Bensimon 2016
D283 Medulloblastoma in organ culture	Human			9.8	7.2	29.6	Rubinstein 1987
Malignant glioma	Human			7.0-10.0			Rubinstein 1987
HeLa S3	Human			6		25	Cited by Cameron&Greulich 1963
HeLa	Human			8.5		28	Cited by Cameron&Greulich 1963
Epithelioma	Human		21	12	5	38	Frindel E 1968
Leukemia	Human		36	26	4	66	Saunders E F 1967
Melanoma	Human		37	21	5	63	Shirakawa 1970
Squamous cell carcinoma Human	Human		28.8	12.5	6.9 (G2/M)	50.2	Friedland & Weinstein 1977
MEFs	Mouse	6.34		8			Fereira 2017
mESCs	Mouse	6.34	2	5	2		Fereira 2017
Embryonic Striatum progenitor	Mouse	6.34		5			Bhide 1996
Esophagus	Mouse	6.34		6.9		181	Greulich 1963
Tongue	Mouse	6.34		7		40	Greulich 1963
Duodenum	Mouse	6.34		7.4		18.5	Greulich 1963
Lower Ileum	Mouse	6.34		7.1		16.7	Greulich 1963
Colon	Mouse	6.34		7.5		32.6	Greulich 1963
Primary Spermatocyte AII	Mouse	6.34	7.5	8			Monesi 1962
Primary Spermatocyte AIII	Mouse	6.34	8	7			Monesi 1962
Primary Spermatocyte AIV	Mouse	6.34	9.5	5.0-6.0			Monesi 1962
Primary Spermatocyte Intermediate	Mouse	6.34	8.5	12.5	3.0-4.0		Monesi 1962
Primary Spermatocytes B	Mouse	6.34	10.5	14.5	3.0-4.0		Monesi 1962

Ileum	Mouse	6.34		7.5		18.0-19.0	Cited by Cameron&Greulich 1963
Hair follicle	Mouse	6.34		7.5		12	Cited by Cameron&Greulich 1963
Breast cancer	Mouse	6.34		10		24.0-72.0	Cited by Cameron&Greulich 1963
Tongue	Mouse	6.34		8.5		96	Cited by Cameron&Greulich 1963
Neural tube	Mouse	6.34	3	4	1.5	8.5	Kauffman S L 1966
Primitive ependymal cells	Mouse	6.34	4	5.5	1.5	11	Atlas M & Bond V P 1963
Mesenchymal cells	Mouse	6.34	13	5.5	1.5	20	Atlas M & Bond V P 1963
Primitive erythroblasts	Mouse	6.34		11	1.5		Atlas M & Bond V P 1963
Corneal epithelium	Mouse	6.34		8	5	100	Epifanova O I 1966
Esophagus	Mouse	6.34		2	8	30	Greulich 1964
Forestomach	Mouse	6.34	15	13.5	2	30	Wolfsberg M 1964
Duodenum	Mouse	6.34	3	7.0-11.0	1.3	10.5-13.0	Lesher S 1961
Colon Mouse	Mouse	6.34	16	7	1	24	Lipkin & Quastler 1962
Liver parenchymal cells Mouse	Mouse	6.34		8	4		Fabrikant J I 1967
Bone marrow all cells	Mouse	6.34	2	4.5	2	8.5	Frindel E 1967
Bladder epithelium	Mouse	6.34	10	6	3	19	Levi P E 1969
Testis spermatogonia	Mouse	6.34	7.5-10.5	7.5-18	3.0-8.0	26.0-31.0	Monesi V 1962
Uterine epithelium	Mouse	6.34	32	8	2	42	Epifanova O I 1963
Vaginal epithelium	Mouse	6.34		7.5	2		Thrasher J D 1967
Mammary gland alveoli	Mouse	6.34		9.0-28.0	2		Bresciani F 1965
Epidermis basal cells	Mouse	6.34		7	1	30	Devik F 1962
Ear	Mouse	6.34		30	7		Sherman F G 1961
Hair follicle Mouse	Mouse	6.34	3	7	2	12	Cattaneo S M 1961
Hair follicle Ref 1 Mouse	Mouse	6.34	4.5	6	2	12.5	Griem M L 1966

Adenocarcinoma Mouse	Mouse	6.34	22	10	3	35	Mendelsohn M L 1960
Ehrilch ascite tumor	Mouse	6.34	0.2	13	3	16	Lala P K 1966
Fibrosarcoma NCTC 2472	Mouse	6.34	4.9	10.4	1.7	17	Frindel E 1967
Sarcoma 180	Mouse	6.34	2.6	8.7	2.5	14	Simpson Herren L & HH Lloyd 1970
Neocortical proliferative zone of fetal mouse	Mouse	6.34	9.3	15.1	3.8		Takashai 1993
C2C12	Mouse	11.4	8.8	9.4	3.7	22.6	Cardoso & Bensimon 2016
pmel-1 Mice CD8 T cells	Mouse	6.34	1	4.7	0.3 (G2/M)		Norie Sugitani 2022
pmel-1 Mice Fibroblasts	Mouse	6.34	13	8.2	7.5 (G2/M)		Norie Sugitani 2022
pmel-1 Mice B16 cells	Mouse	6.34	7.1	9.3	0.7 (G2/M)		Norie Sugitani 2022
Ventricular zone Pax6+ Tbr2-	P1 Ferret	4.82	36.1	16.1	2	55.5	Garcia 2016
Ventricular zone Pax6+ Tbr2+	P1 Ferret	4.82	36.3	19.7	2	58.5	Garcia 2016
Inner subventricular zone Pax6+ Tbr2-	P1 Ferret	4.82	37.7	8.6	2.2	49.4	Garcia 2016
Inner subventricular zone Pax6+ Tbr2+	P1 Ferret	4.82	28.6	9.4	2	42	Garcia 2016
Inner subventricular zone Pax6- Tbr2+	P1 Ferret	4.82	39.8	1.9	1.7	45.8	Garcia 2016
Outer subventricular zone Pax6+ Tbr2-	P1 Ferret	4.82	45	22.6	2.1	71.3	Garcia 2016
Outer subventricular zone Pax6+ Tbr2+	P1 Ferret	4.82	32.4	9.7	1.9	45	Garcia 2016
Outer subventricular zone Pax6- Tbr2+	P1 Ferret	4.82	35.6	3.1	1.7	41.3	Garcia 2016
Embryonic midbrain	Rat	6.57		7.5-8.6		15	Faustman 2003

Squamous cell carcinoma Rat	Rat	6.57	15	9	3		Lane 1980
Tracheal Epithelium	Rat	6.57		8.0-9.0	2.5-3.5		Lane 1976
Hepatocyte	Rat	6.57		8			Cited by Cameron&Greulich 1963
Chondrocyte	Rat	6.57		8.5			Cited by Cameron&Greulich 1963
Metaphsis	Rat	6.57		8		36	Cited by Cameron&Greulich 1963
Endostcum	Rat	6.57		8		57	Cited by Cameron&Greulich 1963
Periosteum	Rat	6.57		8		114	Cited by Cameron&Greulich 1963
Jejunum	Rat	6.57	2.5	6.5	1.5	10.5	Cairnie A B 1965
Liver parenchymal cells	Rat	6.57	9	9	3.5	21.5	Post J & J Hoffman 1964
Tracheobronchial epithelium	Rat	6.57		7	3.5		Wells A B 1970
Erythroblasts 6 week	Rat	6.57	2	4.9-5.0	2	9	Roylance P J 1968
Erythroblast 11-13 week	Rat	6.57	1.5	7.5	1.5	10.5	Hanna 1969
Newborn Cartillage cells	Rat	6.57	6.8	11.6	4.6	22	Dixon B 1971
6 day old osteoprogenitor cells	Rat	6.57	14	8	2	24	Young R w 1962
Adenocarcinoma	Rat	6.57	14	9	1	24	Simpson Herren L & HH Lloyd 1970
Adenosarcoma BICR/M1	Rat	6.57	7.5	7.5	2.7	18	Steel G C 1966
Fibrosarcoma RIB5	Rat	6.57	4	8	1.6	13	Denekamp 1970
Rhabomyosarcoma	Rat	6.57	6.2	9.7	2.7	19	Hermens A F & G W Barendsen 1967
Hair follicle	Sheep	5.22	9.4	9.5	1.6	21	Downes A M 1966
Armadillo triticale (Hexaploid)	*Triticum + Secale* (wheat+rye)		2.77	5.25	2.5	11	Kaltsikes 1972

Rosner triticale (Hexaploid)	*Triticum + Secale* (wheat+rye)		2.4	6.25	3.1	12	Kaltsikes 1972
A-6 Xenopus somatic cell line 18 degrees	*Xenopus laevis*	6.2	30.2	29.5	10.1		A. Al-Saleh 1984
A-6 Xenopus somatic cell line 23 degrees	*Xenopus laevis*	6.2	15.1	16.2	4.3		A. Al-Saleh 1984
A-6 Xenopus somatic cell line 28 degrees	*Xenopus laevis*	6.2	12	13.8	3.9		A. Al-Saleh 1984
S. cerevisiae	Yeast	0.0121		0.283			Amnon Koren 2010
Arabidopsis	Arabidopsis	0.13		1.9			Arabidopsis Genome Initiative, 2000
Rice cv. Nipponbare Roots	Rice cv. *Nipponbare*	0.39		1.9			Matsumoto et al., 2005
Rice cv. Nipponbare Culture	Rice cv. *Nipponbare*	0.39		4.7			Matsumoto et al., 2005
Dicotyledon root tip meristem of diploid cells	*Linum usitatissimum*	1.37		4.1		11.2	Evans & Rees 1971
Maize cv. B73	Maize cv. B73	2.3		3.9			Schnable et al., 2009
Dicotyledon root tip meristem of diploid cells	*Lycopersicum esculentum*	3.81		4.3		10.6	Evans & Rees 1971
Dicotyledon root tip meristem of diploid cells	Crepis capillaris	4.11		3.25		10.8	Evans & Rees 1971
Barley cv. Morex	Barley cv. Morex	5.1		2.7			Mayer et al., 2012
Dicotyledon root tip meristem of diploid cells	*Lathyrus angulatus*	8.70	4.4	3.9		12.25	Evans & Rees 1971
Monocotyledon root tip meristem of diploid cells	*Lolium perenne*	9.68	0.5	4.2		8.6	Evans & Rees 1971
Monocotyledon root tip meristem of diploid cells	*Zea mays*	10.8	0.5	4.25		10.5	Evans & Rees 1971
Dicotyledon root tip meristem of diploid cells	*Lathyrus articulatus*	12.1	3.9	4.3		14.25	Evans & Rees 1971

Wheat cv. Chinese Spring	Wheat cv. Chinese Spring	17		2.9			Brenchley et al., 2012
Dicotyledon root tip meristem of diploid cells	*Lathyrus tingitanus*	17.7	6.3	5.3		16.75	Evans & Rees 1971
Monocotyledon root tip meristem of diploid cells	*Secale cereale*	18.5	1	6		11.7	Evans & Rees 1971
Dicotyledon root tip meristem of diploid cells	*Lathyrus hirsutus*	19.7	7.8	5		18	Evans & Rees 1971
Dicotyledon root tip meristem of diploid cells	*Nigella damascena*	20.6	1.5	10.5		16.5	Evans & Rees 1971
Dicotyledon root tip meristem of diploid cells	*Vica faba*	23.4	4.9	7.3		19.3	Evans & Rees 1971
Monocotyledon root tip meristem of diploid cells	*Allium fistulosum*	25.7	<0.5	7.1		15.1	Evans & Rees 1971
Monocotyledon root tip meristem of diploid cells	*A. cepa * A. fistulosum*	29.2		8.6		16.6	Evans & Rees 1971
Monocotyledon root tip meristem of diploid cells	*Allium cepa*	32.76	<0.5	9.6		17.8	Evans & Rees 1971
Monocotyledon root tip meristem of diploid cells	*Tradescantia paludosa*	37.9		10.8		20	Evans & Rees 1971
Monocotyledon root tip meristem of diploid cells	*Hyacinthus orientalis*	48.6	0.8	13.6		24	Evans & Rees 1971
HCT-116	Human		5.4	6.8	3.8	16	Fereira 2017
L-strain cells	L-strain cells			6.0-7.0		20	Cited by Cameron&Greulich 1963
Hamster cells	Hamster cells			6		14	Cited by Cameron&Greulich 1963
Ascites tumor cells	Ascites tumor cells			7.5		18	Cited by Cameron&Greulich 1963

Liver cells	Liver cells			8		31	Cited by Cameron&Greulich 1963
L-5178Y ascites	L-5178Y ascites			6.9-7.4			Cited by Cameron&Greulich 1963
BNL tumor	BNL tumor			8		16	Cited by Cameron&Greulich 1963
Tradescantia paludosa	*Tradescantia paludosa 2N*			10.7			Troy & Wimber 1969
Tradescantia paludosa	*Tradescantia paludosa 24N*			10.4			Troy & Wimber 1969
Ornithogatum virens	*Ornithogatum virens 2N*			7.9			Troy & Wimber 1969
Ornithogatum virens	*Ornithogatum virens 4N*			7.9			Troy & Wimber 1969
Cymbidium	*Cymbidium* 2N			7.1			Troy & Wimber 1969
Cymbidium	*Cymbidium* 4N			7.7			Troy & Wimber 1969
Lycopersicum esculentum	*Lycopersicum esculentum* 2N			7.2			Troy & Wimber 1969
Lycopersicum esculentum	*Lycopersicum esculentum* 4N			7.6			Troy & Wimber 1969
Chrysanthemum cozymbosum	*Chrysanthemum cozymbosum*			5.9			Troy & Wimber 1969
Chrysanthemum yezoense	*Chrysanthemum yezoense*			4.8			Troy & Wimber 1969
Chrysanthemum arelieum	*Chrysanthemum arelieum*			5.1			Troy & Wimber 1969
Chrysanthemum lacustre	*Chrysanthemum lacustre*			6.3			Troy & Wimber 1969
Caulobacter crescentus	*Caulobacter crescentus*		0.5	1.3	0.3	2.5	Laub 2000
Escheria coli (Fast Growth)	*Escheria coli*	0.0046		0.6	0.3		Helmstetter &Cooper 1968
Bacillus subtilis (37oC)	*Bacillus subtilis*	0.00421		0.9			M Sharpe 1998
S. solfataricus	*S. solfataricus*	0.0031		2.6	4.25	7	Bernander 1997
S.acidocaldarius	*S.acidocaldarius*	0.0027		0.9-1.4	2.1-2.55	3.55	Bernander 1997
Phaeodactylum tricornutum	*Phaeodactylum tricornutum*	0.0274	9.4	1.4	7.6	18.7	Brzezinski 1990

Cylindrotheca fusiformis	*Cylindrotheca fusiformis*		9.4	1.1	4	16.2	Brzezinski 1990
Thalassiosira weissflogii	*Thalassiosira weissflogii*	16.87	4.5	3.6	2.1	13.3	Brzezinski 1990
Thalassiosira pseudonana	*Thalassiosira pseudonana*	0.0324	6	0.3	3.1	9.8	Brzezinski 1990
Chaetoceros muellerii var. *subsalsum*	*Chaetoceros muellerii var. subsalsum*		12.9	0.9	1.9	16.2	Brzezinski 1990
Minutocellus polymorphus	*Minutocellus polymorphus*	1.86	10.6	1.3	0.5	13.9	Brzezinski 1990
Chaetoceros simplex	*Chaetoceros simplex*		7.8	0.8	0.3	9.6	Brzezinski 1990

	**Source**
Dog Genome Size	Hoeppner, M. P 2014
Drosophilla melanogaster Genome Size	Adams 2000
Human Genome Size	Piovesan 2019 - average of male and female
Sheep Genome Size	Yu Jiang 2014
Xenopus laevis Genome Size	U Hellsten 2010
S. cerevisiae Genome Size	Genome size - Belda 2019
Escheria coli Genome Size	Blattner 1997
Thalassiosira weissflogii Genome Size	Connolly 2008
Minutocellus polymorphus Genome Size	Connolly 2008
Arabidopsis Genome Size	Arabidopsis Genome Initiative, 2000
Conversions from picogram to Gbp	Dolezel 2003
